# Methanolic extract of *Euchelus asper* exhibits in-ovo anti-angiogenic and in vitro anti-proliferative activities

**DOI:** 10.1186/s40659-017-0147-2

**Published:** 2017-12-12

**Authors:** Sweta Agrawal, Sachin Chaugule, Shashank More, Gargi Rane, Madhavi Indap

**Affiliations:** Central Research Laboratory, D. G. Ruparel College, Senapati Bapat Marg, Mahim, Mumbai, 400 016 India

**Keywords:** Marine bioactives, *Euchelus asper*, Mollusc, Anti-angiogenesis, Anti-proliferative, Anti-cancer, CAM, Chorio-allantoic membrane

## Abstract

**Background:**

The marine environment is a rich source of bioactive natural products. Many of the marine bioactive compounds have been derived successfully from molluscs. *Euchelus asper* is a marine mollusc which is commonly found in the intertidal rocky regions of the Mumbai coast. The present study was focused on evaluating the anti-angiogenic and anti- proliferative activities of methanolic extract of *Euchelus asper* (EAME).

**Methods:**

The anti-angiogenic activity of EAME (50–800 μg/mL) was assessed by chick chorio-allantoic membrane (CAM) model wherein multiple parameters in the CAM blood vessels were analysed through morphometric and histological investigations. In vitro testing of EAME (5–20 μg/mL) included its cytotoxicity against three different cancer cell lines, its effect on cell proliferation by wound healing assay as well as their relevant molecular mechanisms. Statistical analysis was carried out by two-tailed student’s t test for two unpaired groups.

**Results:**

Analysis of CAM revealed that the extract is effective in reducing the branching points of the 1st order blood vessels or capillaries of CAM. Histological analysis of CAM showed significant decrease in capillary plexus and compartmentalization along with increase in mesodermal blood vessels, thus establishing its anti-angiogenicity. Further, EAME exhibited moderate but significant cytotoxicity against A549 non-small cell lung carcinoma cell line. We also demonstrated that the cytotoxicity of EAME in A549 was associated with its apoptotic activity by subG1 phase arrest. Lastly, EAME significantly reduced A549 proliferation by reducing the expression of Matrix metalloproteinase-2 (MMP-2) and Matrix metalloproteinase-9 (MMP-9).

**Conclusion:**

Overall, our study suggested that EAME has potential to inhibit tumour angiogenic and proliferative activity and may be a potential source for development of new anti-cancer pharmaceuticals.

## Background

Angiogenesis is a physiological process involving growth of nascent blood vessels from the pre-existing vasculature. It is a complex process, regulated by the balance between pro- and anti-angiogenic factors. Interference in regulation of angiogenesis leading to excessive blood supply is associated with diabetic retinopathy, inflammatory diseases as well as cancer growth [1]. Thus, discovery of novel anti-angiogenic agents could be helpful in the development of anti-angiogenic therapy against such diseases. Since solid tumours cannot grow beyond 1–2 mm in size without angiogenic supply; therefore anti-angiogenic therapy is emerging as a powerful tool in the quest to eradicate tumours.

In the recent years, extensive efforts have been taken to target angiogenesis for cancer therapy. As a result, several anti-angiogenic drugs are currently used to treat various malignancies, targeting one or more angiogenic factors. It is becoming alarmingly clear that available anti-angiogenic mono-therapies are not meeting their expectations of “starving the tumour”. One of the important reasons for this is the fact that as tumour hypoxia increases so does tumour invasiveness and metastasis. The other important reason behind failure of anti-angiogenic mono-therapy is the acquired resistance in tumours [2]. On the other hand, traditional chemotherapeutic agents that kill cancer cells directly are associated with several acute side-effects leading to relapse and metastasis. In such situations, a combination strategy involving use of anti-angiogenic agents along with other therapies that target possible resistance mechanisms have proven to be a more efficient anti-cancer therapy [3]. Clinical studies have shown that patients on anti-angiogenic therapy, especially those on combination therapy with chemotherapy, may have better survival rate than those on chemotherapy alone [4]. Such data strongly supports the belief that the future of anti-angiogenic therapy lies in combination with chemotherapy.

Since last few decades, extensive work has been carried out in the field of cancer research. As most of the synthetic anti-angiogenic drugs come with limitations like narrow adaptation range and associated side-effects, researchers are increasingly turning towards natural sources as raw material for such drugs since many of them are included in diet and so they have lesser side-effects. Various compounds with anti-cancer properties have been derived from either plants or terrestrial microbes, both of which are known for their uses in the treatment of many diseases since historical times. For marine organisms this is not the case, as the marine bioactives were first isolated in the early 1950s [5]. The marine environment consists of a vast chemical diversity due to its rich biodiversity. Bioactive compounds isolated from marine organisms till date, have shown a variety of pharmacological properties including anti-tumour, anti-angiogenic, anti-proliferative, cytotoxic, anti-inflammatory, anti-oxidant as well as antibiotic and antifouling [6]. Although, bioactives have been isolated from several phyla present in the sea, still a vast majority of oceanic biodiversity remains under-explored.

Molluscs are the second largest phylum among the marine life and are found to be promising candidates for drug discovery and development. As many molluscs are a part of human diet and are also known for their uses in ethno-medicine, they provide very good raw material for drug development. Several anti-cancer agents are unearthed from cephalopods, bivalves as well as gastropods. *Euchelus asper* is one such commonly found mollusc along the west coast of India. Previous studies on this organism, has revealed that its ether soluble fraction (EAE) exerted immuno-stimulatory activity in vitro by phagocytosis [7]. It has also shown immunosuppressant activity in vivo by plaque formation assay [8]. Recently, methanolic extract of *E. asper* was discovered to possess anti-osteoporotic activity in vivo [9]. Till date, there was lack of scientific reports available on anti-angiogenic activity of marine molluscs which encouraged us to evaluate anti-angiogenic and anti-proliferative activity of *Euchelus asper* methanolic extract for its probable use in cancer therapeutics.

## Methods

### Extraction of *Euchelus asper*

*Euchelus asper* was collected from rocky sea shore of Mumbai, India. Organism was deshelled and inner body mass was weighed and cold percolated in methanol in a proportion of 1:5. The extraction was repeated three times, each time adding fresh methanol. Recovery of methanol from the extract was carried out by vacuum distillation. The crude extract termed as EAME and was stored at 4 °C for further use.

### Chick chorio-allantoic membrane assay

#### Preparation of EAME concentration

Different concentration of crude EAME extracts ranging from 50 to 800 µg/mL for in vivo CAM assay were prepared diluting it with Hank’s balanced salt solution (HBSS). HBSS was used as normal control and 60 µg/10 µL of heparin in hydrocortisone was used as positive control [10].

#### Anti-angiogenesis assay using chick CAM model

Modified chorio-allantoic membrane assay (CAM assay) was performed on 5 days incubated hen eggs [11]. In short, fertilized white Leghorn chicken eggs were obtained from Central Poultry Development Organization, Mumbai. Eggs were cleaned with a 70% alcohol solution and incubated at 37–38 °C with approximately 75–80% humidity. On day four, 2–3 mL of albumin was removed to allow detachment of membrane from shell. On day five, chick embryos were exposed by making a 20 mm window in the egg shell and 200 µL of HBSS containing different concentrations of extract was added under sterile conditions. Simultaneously, normal (HBSS) and positive controls (Heparin–Hydrocortisone) were also maintained to compare the results. After 48 h, the eggs were sacrificed and various parameters were tested for angiogenic response.

#### Morphometric analysis

Multiple images of each CAM were taken using digital compound microscope, Kyowa (Model no—10151). For each concentration, average readings from 10 eggs were taken. Various morphometric parameters like CAM area, weight of embryo, average number of branching points and number of first order blood vessels were studied. Non-parametric criteria like structural distortions in the normal pattern of blood vessel branching and morphology were also evaluated.

#### Histological analysis

A portion of CAM that included first order blood vessels was fixed in 10% neutral formalin. These CAM were further processed for standard histological preparation and stained with Ehrlich’s Hematoxylene and Eosin [12].Histological quantification was carried out using digital microscope and Idn2 software. 8–10 images of each slide were captured and used for quantification. Each image was of 0.094 mm^2^ area. Percent of ectoderm subtended by capillary plexus, CAM width, density of mesenchymal cells and mesodermal cells, length and width of capillaries were considered for measuring the anti-angiogenic response.

### Cell line culture

Cancer cell lines A549 (lung carcinoma), MCF7 (breast carcinoma) and HeLa (ovarian cancer cell line) were procured from National Centre for Cell Sciences (NCCS), Pune, India. The A549cell line was maintained in F12—Ham’s medium (Hi-Media) supplemented with 10% FBS (Hi-media, India), and penicillin and streptomycin solution. HeLa was cultured in DMEM (Hi-media) supplemented with 10% FBS, 1% pen-strep and 2% l-glutamine solution whereas MCF7 cell line was grown in RPMI-1640 (Hi-Media) medium supplemented with 10% FBS and 1% penicillin and streptomycin solution. These cell lines were maintained in sterile T-flasks (BD Falcon) at 37 °C in a humidified atmosphere with 5% CO_2_. Cells were passaged and used for further experiments at 80% confluent state.

### Sulphorhodamine-B (SRB) assay

Cytotoxicity of EAME was tested on three cell lines, MCF7, A549 and HeLa using Sulphorhodamine-B (SRB) assay [13]. In brief, exponentially growing cells were seeded into 96-well plates at 5 × 10^3^ cells/well and allowed to adhere for 18–24 h. Then cells were treated with various concentrations of EAME (5–20 μg/mL) along with growth media and incubated for 24, 48 and 72 h. Control wells received only growth medium. After the incubation period, 50 µL of 50% trichloroacetic acid was added without disturbing the medium and the plates were maintained at 4 °C for 60 min. Plates were then washed five times with distilled water and stained with 4% SRB dye for 30 min. After the incubation, plates were quickly washed with 1% acetic acid several times to remove the unbound dye, and allowed to dry. 100 µL tris-base was added to each well and after 10 min, the plates were shaken. SRB bound to protein was measured by absorbance at a 492 nm wavelength using a Bio-Rad Elisa plate reader (iMark).

### Cell cycle analysis by flow cytometry

Cell cycle analysis was performed on A549 cell line using Flow cytometry as described by Riccardi et al. [14]. Cells were seeded in 24-well plates (2 × 105/well) and cultured with or without EAME at different concentrations. After 24 h, cells were harvested using 0.3% trypsin–EDTA (ethylenediamine tetra-acetic acid) solution, washed with ice cold phosphate buffer saline (PBS), centrifuged and fixed in 70% ice cold ethanol. The fixed cells were then rehydrated in PBS, centrifuged and re-suspended in PBS 300 µL containing 100 µg/mL RNAse A and stained with 100 µL propidium iodide (40 µg/mL).

Apoptosis and cell cycle distribution of EAME treated A549 cells were assessed using a BD FACS Calibur flow cytometer (BD Biosciences) using CellQuest Software. Flow cytometric analysis of DNA content was based on the acquisition of 10,000 events and the percentages of cells in different phases of the cell cycle were analysed using the ModFit LT™ program. Assays were carried out in triplicate, and the results are representative of three independent experiments.

### Scratch wound healing assay

For determination of cell migration and proliferation, scratch wound healing assay was performed as described by Liang et al. [15]. For this assay, A549 cells were seeded into 24-well tissue culture plate at a density 5 × 10^5^ cells/well and allowed to grow to a monolayer. The monolayer was then scratched with micro-pipette tip twice in perpendicular fashion and the medium was replaced by fresh medium containing treatment (EAME 5–20 μg/mL). Control wells were fed with only growth medium. The scratch wound was monitored and photographed at 0 h with a (Evos XL core) inverted microscope. After 24 h of incubation, the cells were stained with 1% crystal violet in 2% ethanol for 30 min and photographed. At least 4 photos of random fields were captured per well and the experiment was performed in triplicate. The area of wound remaining after 24 h in treated and control samples was compared in order to check the anti-proliferative activity of EAME.

### Gelatin zymography

The enzymatic activity of matrix metalloproteinase MMP-2 and MMP-9 in the conditioned medium of A549 cells was determined using gelatin zymography [16]. The conditioned medium was resolved by 8% SDS-PAGE containing 0.1% gelatin after A549 cells were treated with various concentrations (0, 5, 10 and 20 μg/mL) EAME for 24 h. After electropho-resis, gels were washed with 2.5% Triton X-100 solution, and then incubated in developing buffer (50 mM Tris–HCl pH 7.8, 5 mM CaCl_2_, 0.2 M NaCl and 0.02% Brij-35) for 48 h at 37 °C. Then the gels were stained with Coomassie brilliant blue R-250. Clear bands on blue zymogram representing gelatinolytic activity were quantified by densitometer measurement using digital imaging analysis software Image-J.

### Statistical analysis

The data are expressed as mean ± standard error (SE). Statistical analysis was done by the two-tailed student’s t test for two unpaired groups. Differences with p values of less than 0.05 were considered statistically significant.

## Results

*Euchelus asper* was collected in bulk from the shores of Mumbai region and extracted in methanol yielding 1.25% of extract which was termed EAME. Preliminary chemical investigation of EAME showed presence of primarily proteins and amino acids as well as some amount of carbohydrate and fats.

**Fig. 1:**
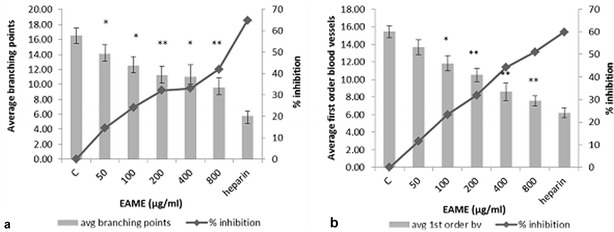
Anti-angiogenic effect of EAME **a** represents effect of EAME on average branching points of capillaries (1st order blood vessels). **b** Represents the dose-dependent response of EAME on the number of first order blood vessels of CAM. Each group contained at least 10 eggs. Each bar represents the mean ± SE of the three independent experiments. *p < 0.05; **p < 0.001 compared with the control

The effect of EAME on CAM blood vessels was assessed by carrying out morphometric as well as histological analysis of CAM blood vessels. Total number of branching points and the number of first order blood vessels in CAM images were counted as a part of morphometric analysis. As shown in the Fig. 1a, b, EAME showed a dose dependent decrease in the branching points as well as first order blood vessels. At highest concentration 800 µg/mL, 42% (p < 0.001) branching point inhibition and 51% (p < 0.001) reduction of first order blood vessels was observed. The anti-angiogenic response of heparin–hydrocortisone was highest where the branching points reduced up to 64%, as it is a known anti-angiogenic drug.

Area of CAM and weight of embryo were measured after treatment in order to see whether EAME has any toxic effect on the embryo. The weight of chick embryo did not reduce on treatment with EAME; however area of CAM at highest concentration (9.571 ± 0.579 cm^2^) was reduced as compared with that of HBSS control (10.825 ± 0.415 cm^2^).Overall, all treated samples showed moderate decreased in CAM area as compared to HBSS control (Table 1).

**Table 1:** showing the effect of EAME on chick embryo weight and area of CAM

Effect of EAME on chick embryo		
Group	Average weight of embryo	Average area of CAM
Control	0.727 ± 0.050	10.825 ± 0.415
50 μg/mL	0.746 ± 0.026*	8.995 ± 0.479*
100 μg/mL	0.767 ± 0.029**	9.039 ± 0.334*
200 μg/mL	0.735 ± 0.035**	8.442 ± 0.666
400 μg/mL	0.733 ± 0.021	9.640 ± 0.576*
800 μg/mL	0.727 ± 0.032	9.571 ± 0.579*
Heparin	0.621 ± 0.064**	8.432 ± 1.06

* represents p ≤ 0.05; ** represents p ≤ 0.001, p values in comparison with control

**Fig. 2:**
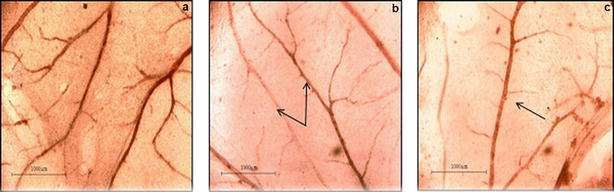
CAM images showing effect of EAME on morphology of blood vessels (40× magnification, scale represents 1000 μm). **a** HBSS control (tree-like branching pattern); **b**, **c** treated with EAME 800 µg/mL; **b** arrow indicates parallel effect; **c** arrow indicates abnormal thinning of blood vessels

Imaging analysis of treated and control CAM disclosed marked distortions in the normal pattern of blood vessels branching and morphology. In blind evaluations, most of the control sections were distinctly different from the treated ones. Moreover, some specific abnormalities in the blood vessel pattern like disorganization of pattern of branching (“disorganize”) and increased occurrence of blood vessels running parallel to each other without branching (“long and parallel”), were observed in treated CAMs as shown in Fig. 2. Lastly, the treated CAMs displayed fewer and thinner blood vessels than control.

**Fig. 3:**
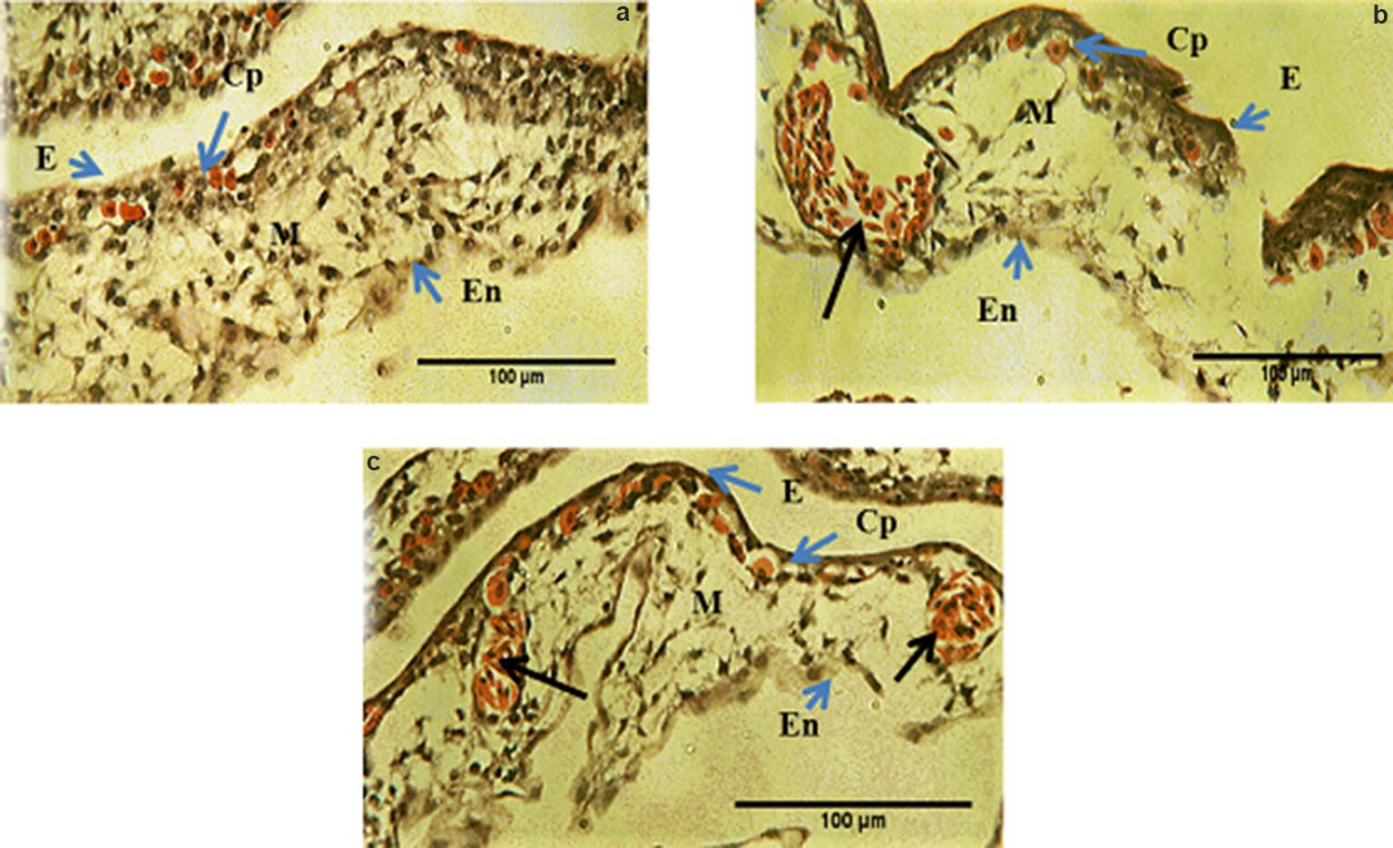
Histological images of CAM (400× magnification, scale represents 100 μm) showing capillary formation in treated and control CAMs. **a** Control image showing proper capillary plexus (CP) in the ectoderm (E), mesoderm (M) and no mesodermal blood vessels followed by endoderm (En). **b** Treated (EAME 800 µg/mL) showing decrease in capillary plexus (CP), presence of mesodermal blood vessel (black arrow) and somewhat abnormal ectoderm (E). **c** Treated (heparin-hydrocortisone) showing reduced CAM thickness, more number of mesodermal blood vessels (black arrow), reduced capillary plexus (CP)

**Fig. 4:**
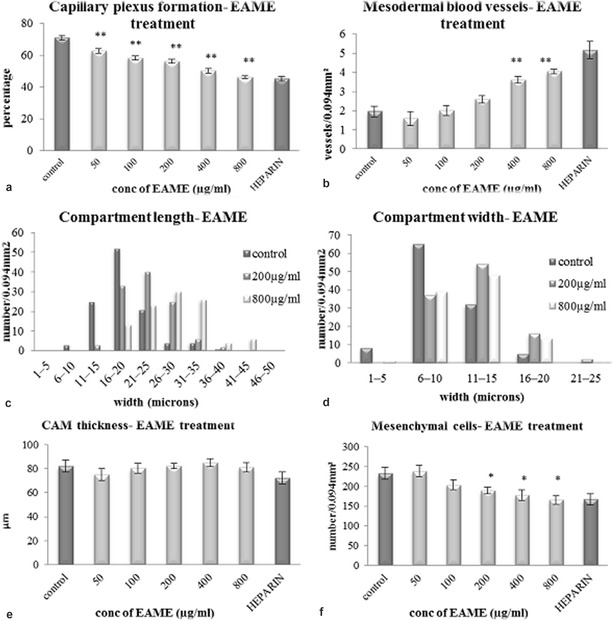
Effect of EAME on the different cellular aspects of CAM represented by histology. **a** Capillary plexus subtended by ectoderm. **b** Number of mesodermal blood vessels. **c** frequency of capillary length. **d** Frequency of capillary width. **e** Thickness of CAM and **f** Number of mesenchymal cells. Each bar represents the mean ± SE of the three independent experiments. (* represents p ≤ 0.05; ** represents p ≤ 0.001 in comparison with control)

The formation of capillary plexus in CAM occurs rapidly during the 5th and the 6th day of incubation [17]. To determine whether EAME affected the process of capillary formation, detailed analysis of histological sections of treated and control CAM was carried out. It was found that HBSS control showed normal capillary plexus formation beneath the ectoderm. In contrast, the CAMs treated with EAME and heparin showed very little capillary plexus beneath the ectoderm as showed in Fig. 3. While control sections showed an average 70% of ectoderm covered by capillary plexus, this area reduced to 46% when treated with EAME (800 µg/mL) and 45% when treated with heparin control (Fig. 4a). As compared to HBSS control, the number mesodermal blood vessels in CAMs treated with EAME or heparin were quite high (Fig. 4b) indicating that they most likely failed to migrate towards the ectoderm.

In order to determine whether, EAME affects the subdivision of secondary and tertiary blood vessels, we measured the length and width of compartments of blood vessels. In case of control CAMs frequency of capillaries with shorter length (11–20 microns) and smaller width (6–10 microns) was highest. As the concentration of EAME increased, the CAMs were dominated by capillaries of greater length and width. Thus, in CAMs treated with 800 µg/mL EAME, most of the capillaries were of length more than 26 microns (Fig. 4c) and width more than 11 microns (Fig. 4d). This increase in compartment length and width along with increase in mesodermal blood vessels suggest that EAME must be affecting the compartmentalization during intussusceptive angiogenesis.

CAM thickness and density of mesenchymal cells were observed since capillary plexus formation is accompanied by decrease in CAM thickness and mesenchymal cell density. Our results revealed that the thickness of treated as well as control CAMs were not significantly different from each other (Fig. 4e). At the same time, mesenchymal cells in EAME treated CAMs showed slight decrease as compared to the HBSS control (Fig. 4f). These results collectively confirm the significant in ovo anti-angiogenic activity of EAME.

**Fig. 5:**
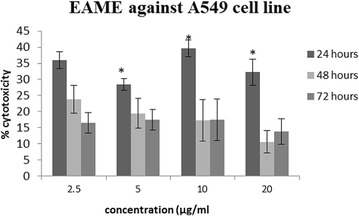
Showing effect of EAME on A549 after 24, 48 and 72 h of treatment. Each bar represents the mean ± SE of the three independent experiments. (* represents p < 0.05 in comparison with control)

Cytotoxicity screening is considered as a basic step in establishing the anti-cancer activity of a substance. To determine whether the anti-angiogenic effect of EAME is associated with its effect on cancer cells, cytotoxicity of EAME was observed in vitro. Initial screening on different cell lines showed that EAME could produce cytotoxic effect against A549 cell line only (Table 2). Therefore, to study this effect further, A549 cell survival was checked for 24, 48 and 72 h of treatment with different concentrations of EAME (0–20 μg/mL). It was observed that EAME produced moderate cytotoxic effect against A549 cell line after 24 h of incubation itself (Fig. 5). Although the cytotoxic effect of EAME was not dose dependent, it exhibited up to 40% (p < 0.05) at 10 μg/mL concentration against A549 cell line. However, this cytotoxicity did not increase progressively after 48 and 72 h treatment which implied that some of the cells may have recovered during the long exposure to treatment.

**Table 2:** Screening of different cell lines for cytotoxicity assessment

Cytotoxicity of EAME against various cancer cell lines			
	HeLa	MCF-7	A549
EAME (20 µg/mL)	−	−	+

(+) means positive cytotoxic activity; (−) means no cytotoxicity

**Fig. 6:**
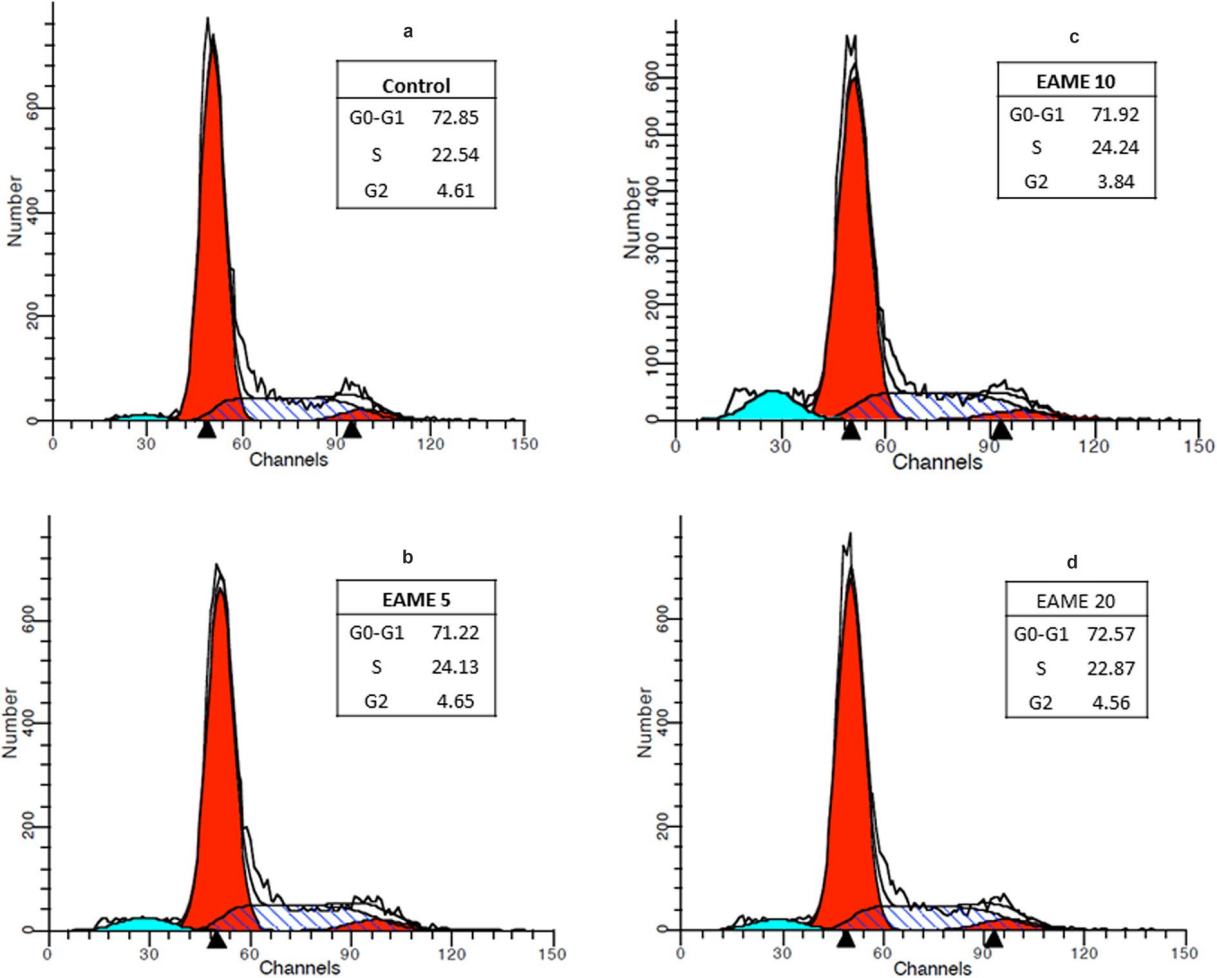
Cell cycle analysis of A549 cells. Cells were cultured without treatment (**a**, control); 5 μg/mL EAME (**b**); 10 μg/mL EAME (**c**) and 20 μg/mL EAME (**d**) for 24 h. The percentage of non-apoptotic cells within each cell cycle was determined by flow cytometry. Compared to the control (**a**), EAME could induce apoptosis (increasing sub-G1 population)

To examine the mechanism of cytotoxicity of EAME on A549 cells, cell cycle distribution was evaluated using flow cytometry as demonstrated in Fig. 6. Table 3 features the percentage of cells in each phase in control and EAME treated cells at 24 h. The sub G1 population represented chromatin degradation associated with apoptosis. When compared to control, a moderate increase was found in the sub G1 group after the cells were treated with 10 μg/mL EAME (8.5%) whereas treatment with 20 μg/mL EAME resulted in a slight increase (4.12%). These results suggest that EAME at 10 μg/mL induces more apoptosis leading to higher cytotoxicity in A549 cells as compared to 20 μg/mL EAME at the same incubation period.

**Table 3:** Effect of EAME on cell cycle distribution analyzed by flow cytometry

	Control	EAME		
		5 μg/mL	10 μg/mL	20 μg/mL
G0/G1	72.85	71.22	71.92	72.57
S phase	22.54	24.13	24.24	22.87
G2/M	4.61	4.65	3.84	4.56
Sub-G1	1.92	4.71*	8.5*	4.12

* represents p ≤ 0.05; ** represents p ≤ 0.001, p values in comparison with control

**Fig. 7:**
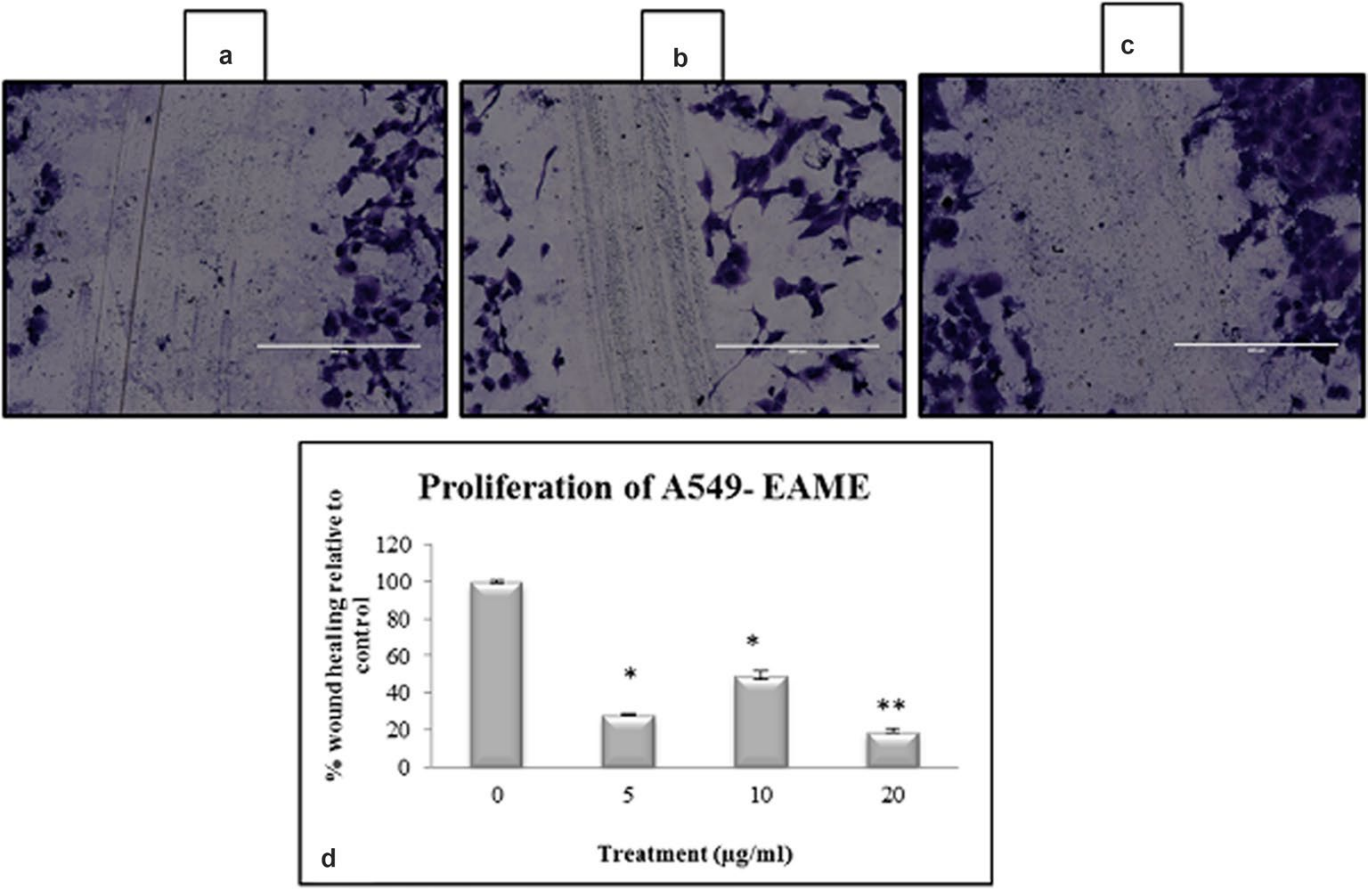
Observations of scratch wound assay on A549 cell line. **a** Represents image of wound area at 0 h (freshly scratched); **b** area of wound healed after 24 h in control; **c** shows effect of EAME (20 μg/mL) on wound closure (scale represents 400 μm length) and **d** graph indicating effect of different concentrations of EAME on wound healing capacity of A549 cell line. The percentage was calculated as relative percent of control considering wound healing in control to be 100%. Each bar represents the mean ± SE of the three independent experiments. (*p < 0.05, **p < 0.001 as compared to control)

The results from scratch wound healing assay revealed that in cells treated with EAME (5, 10 and 20 μg/mL), the percentage of wound healed had greatly reduced compared to control (Fig. 7a–c). The area of wound healed in control was taken as 100% and relative wound healing after EAME treatment was calculated. Thus, EAME at 5 μg/mL concentration reduced the wound area to 28.3% (p < 0.05) of control. This percentage reduced further to 19.43% (p < 0.001) at 20 μg/mL of EAME (Fig. 7d). The above findings indicated that EAME could significantly prevent A549 cells’ migration and proliferation.

**Fig. 8:**
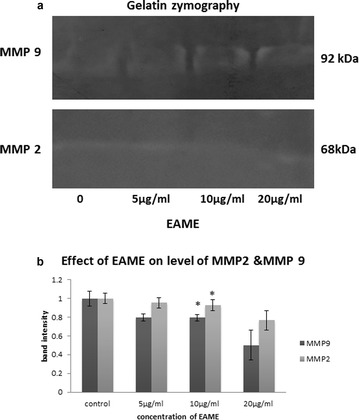
Effect of EAME on the expression of MMP-2/9, in A549 human lung cancer cells. **a** The cells were treated with various concentrations (5, 10, or 20 μg/mL) of EAME for 24 h. The conditioned media were collected and MMP-2 and MMP-9 activities were determined by gelatin zymography. **b** The activities of MMP-2/9 were subsequently quantified by densitometric analysis using Image J software. (* p < 0.05, ** p < 0.001 as compared to control)

To further verify whether EAME inhibited the migration of A549 cells, we investigated the regulatory effect of EAME on MMP-2 and MMP-9 that are the key players in cell migration. As analysed by gelatin zymography (Fig. 8a, b), secretion of MMP-2 and MMP-9 in EAME treated cells reduced significantly as compared to the control cells. The band intensity of both MMP-2 and MMP-9 reduced in a dose dependent manner such that at highest concentration 20 μg/mL EAME, the relative band intensity of MMP-2 and MMP-9 was 0.76 and 0.50 respectively when the band intensity in control samples was considered as 1. Our results confirmed that the anti-metastatic effect of EAME was associated with its inhibitory effect on MMP-2 and MMP-9.

## Discussion

Previous studies on *E. asper* revealed that its ether extract is potent angiogenesis inhibitor; but the study was limited to its qualitative assessment only [18]. In the present study, it is the first time that quantification of anti-angiogenic activity of *E*. *asper* methanolic extract as well as its cytotoxicity was reported.

Molluscs are known for their bioactive compounds and have become a focus of many studies aimed at discovering and isolating novel natural products [19]. In a recent study, Bojnourdi et al. [20] investigated the anti-angiogenic properties of shell extract of the mollusc chiton on CAM model and found that it produced maximum 58% anti-angiogenic activity. In another interesting study by Gupta et al. [21], 22 different marine invertebrate extracts were screened for their anti-angiogenic activity, out of which, *Meretrix meretrix* (47.01% inhibition), *Meretrix casta* (64.63% inhibition), *Telescopium telescopium* (62.02% inhibition) and *Bursa crumena* (60.48% inhibition) showed significant anti-angiogenic activity in CAM. In the present study, EAME showed 42% inhibition of branching points and 51% inhibition of first order blood vessels in CAM (Fig. 1a, b), which is comparable to the work done by Bojnourdi et al. and Gupta et al. However, our analysis was extended further to find the underlying mechanism behind this anti-angiogenic activity by carrying out histological investigations.

The angiogenesis process in CAM is characterized by migration of mesodermal blood vessels, followed by their invasion through basement membrane and extracellular matrix (ECM) and finally their compartmentalization into new capillary vessels in the ectoderm [22]. In normal growth, the number of mesodermal blood vessels decreases as the capillary plexus increases in the early stages of development. It is also known that the formation of capillary plexus in CAM is accompanied by decrease in CAM thickness as well as reduction of mesenchymal cells [23]. This signifies the importance of our present study in which EAME has suppressed CAM angiogenesis by inhibiting migration and compartmentalization of mesodermal blood vessels leading to lesser capillaries subtending the ectoderm as shown in Fig. 4. Since, the embryo weight in treated remained more or less similar to control, it can be said that EAME did not have any adverse effect on the developing embryo and whatever effect found on the blood vessels was solely due to alteration in angiogenesis process. The reduction in area of CAM (Table 1) after treatment was believed to be because of decrease in vasculature.

A similar study carried out by Pandit et al. [10] on the feather star *Lamprometra palmata palmata* revealed the effect of its extract on the histology of CAM. Quantification of CAM histology explained that the capillary plexus in ectoderm decreased along with increase in mesodermal blood vessels and reduction in compartmentalization and after treatment with chloroform fraction of *L. palmata palmata*. However, it was also found that the mesenchymal cell density showed an irregular pattern and the CAM thickness increased with increasing concentration, which is in contrast to our results where both these aspects showed slight decrease after treatment with EAME.

An essential step of angiogenesis is the secretion of matrix metalloproteinases, which degrades the Extracellular membrane (ECM) proteins and allows migration of endothelial cells through the ECM [24]. Around 23 different MMPs are known to be involved in ECM remodelling, out of which MMP-2 and -9 are crucial regulators of angiogenesis and metastasis [25]. Our results from the CAM model showed that EAME efficiently inhibited CAM angiogenesis by increasing the number of mesodermal blood vessels and decreasing the percentage of capillaries in ectoderm. This suggests that the inhibition of CAM angiogenesis by EAME could be a result, in part, from the failure of ECM protein degradation and therefore impede mesodermal blood vessels migration and its subsequent proliferation into capillaries.

In recent years, quite a few studies were carried out to prove anti-angiogenic effect through suppression of MMPs. One such study by Huang et al. [26] demonstrated that *Phyllanthus urinaria* efficiently inhibited embryonic angiogenesis by reducing the MMP-2 activity in the CAM model. Another interesting research conducted by Manjunathan et al. [27] explained the positive angiogenic activity of human recombinant leptin (HRL) in chick CAM model. Their findings verify that the angiogenic response of HRL was due to increase in MMP-2 levels in CAM which caused breakdown of ECM in favour of migration and proliferation of ECs thus supporting the occurrence of sprouting angiogenesis. Since our results are in agreement with these data, it is highly possible that the inhibition of MMP-2 and -9 is one of the reasons for the reduction in capillaries and increase in mesodermal blood vessels in CAM after EAME treatment.

It is already known that angiogenesis plays an important role in development of tumours. Consequently, our results of CAM assay indicated that EAME might be a promising candidate as a new anti-cancer agent. Hence, further studies were conducted to investigate the possible effect of EAME on cancer cell lines directly. We have explored the cytotoxic potential of *E. asper* methanolic extract against three solid tumour cell lines and it was observed that EAME possesses moderate cytotoxicity against A549 cell line.

There are quite a few reports related to combined study of anti-angiogenicity and cytotoxicity in natural products. Baharara et al. [28] reported one such study using extract of the brittle star *Ophiocoma erinaceus.* He demonstrated that brittle star methanolic extract considerably inhibited blood vessels in CAM as well as significantly inhibited A2780 cell growth at 50–100 µg/mL. In another recent study, Mirian et al. [29] determined the in vitro anti-angiogenicity and cytotoxicity of oleo gum resin extract of three plants *Rhus coriaria*, *Pistacia vera* and *Pistacia khinjuk*. However, there are hardly any articles exploring the anti-angiogenic and anti-proliferative effects of mollusc extracts. Our study is probably one of the few that demonstrates these dual activities using a mollusc extract.

Although molluscs have been a good source of cytotoxic compounds, most of these are opisthobranch molluscs (nudibranch, sea hares) while the gastropods remain relatively less explored in this context. A recent study carried out by Antonova et al. [30] reported the significant cytotoxic activity of haemocyanin isolated from two gastropods *Helix aspera* (HaH) and *Helix lucorum* (HlH) against cell lines CAL-29 and T-24 (human bladder carcinoma).Another recent report by Bae et al. [31] focuses on the cytotoxicity of the marine sponge *Haliclona* sp. extracts on A549 cell line. Their results reveal that the *Haliclona* extract, at 100 μg/mL inhibited A549 cell viability by 51.6%. It was also confirmed that this cytotoxic effect was due to its influence on the cell proliferation and cell cycle. Our results indicate that EAME showed 40% inhibition of A549 cell line which is comparable to these studies, therefore it is confirmed that EAME shows moderate but significant cytotoxicity against A549.

The cytotoxic effect of EAME on A549 cells was confirmed through study of cell cycle by flow cytometry. One of the crucial aspects of cell cycle regulation is the DNA structure checkpoints, which arrest the cell cycle at the different phases (G1, S, G2 and M) in response to DNA damage or incomplete replication [32]. Our results demonstrate that EAME moderately increased the amount of Sub-G1 DNA content (Table 3) suggesting that EAME induced apoptosis in the A549 cells. Cancer associated apoptosis-defects play a very important role in drug resistance, so regulation of defective apoptosis has become a fundamental aspect in cancer therapy. Hence, almost all cytotoxic drugs currently used, target on inducing apoptosis in malignant cells [33, 34].

There is increasing evidence of chemo-preventive potential of natural products therefore; continuous efforts are being taken to find anticancer chemotherapeutic drugs from edible and natural sources [35]. Since EAME expressed moderate cytotoxicity in A549, it was attempted to further investigate whether this cytotoxicity is related to its anti-proliferative or anti-migratory effect. Accordingly, the effect of different concentrations of EAME on the wound healing process of A549 cell line was evaluated. Our results have clearly illustrated that EAME significantly reduces the wound healing capacity of A549 cell line, thus establishing its anti-proliferative and anti-migratory property.

Recently many reports illustrate the anti-metastatic effect of various natural or synthetic compounds against A549 non-small cell lung carcinoma. Hsieh, et al. [36] have reported that the anti-proliferative activity of the plant *Kalanchoe tubiflora* extract on A549 cell line was mediated by its induction of cell cycle arrest at G2 phase via its micro-tubule stabilizing property. An individual study on dihydroaustrasulfone alcohol, a compound isolated from marine coral, has illustrated the anti-proliferative activity of this compound on A549 cell line [37]. It was found that the anti-proliferative activity of this compound is correlated with cell cycle arrest at G1 phase, down-regulation of Akt signalling pathway and suppression of proteases MMP-2 and MMP-9.

As explained earlier, MMP-2 and -9 play essential and varied roles in the progression of several types of cancers. A recent report by Webb et al. [38] has revealed the contribution of MMP-2 and MMP-9 in retinoblastoma cells. After comprehensive study, they found that MMP-2 and -9 are involved in stimulating cell migration and contributing to cell viability whereas MMP-9 played an additional role in Angiopoietin-2 production thus enhancing angiogenesis in retinoblastoma cells. Subsequently, a growing number of MMP-2/9 inhibitors are being developed as anti-metastatic agent; Fucoidan, an anti-metastatic agent, significantly reduces lung cancer cell migration and invasion by down-regulating the levels of MMP-2 via the suppression of PI3K-Akt-mTOR and NF-kB signalling pathway [39]. Mere15, novel polypeptide, suppressed the expression of MMP-2 and MMP-9 and eventually inhibits cancer cell metastasis [40]. Our results revealed that EAME significantly reduced the expression of MMP-2/9 in A549 cells (Fig. 8). This result suggested that EAME, similar to other anti-cancer agents, induces anti-metastatic activity by down regulating the expression of MMP-2 and MMP-9.

Owing to its dual targeted anti-cancer property, EAME might be a good source of a novel anti-cancer drug in future. Further work would include fractionating the crude methanolic extract and then testing the individual fractions for the corresponding activities. The molecular mechanism associated with the anti-angiogenic and anti-proliferative activities could be studied then using the active fraction(s).

## Conclusion

In this study, the *E. asper* methanolic extract was found to exhibit in ovo anti-angiogenic function and in vitro anti-proliferative influence on the A549 lung cancer cells. As angiogenesis and cell proliferation are important hallmarks of cancer, the present study elucidates how EAME confers anti-cancer activity against lung carcinoma. Our results have proven that the anti-angiogenic activity exerted by EAME is due to its inhibitory effect on the process of capillary formation in CAM membrane. Likewise, its moderate cytotoxic effect is due to its ability to induce apoptosis in A549 cells. EAME also successfully suppressed the proliferation of lung cancer cells via decreasing the expression of MMP-2/9. The next phase, therefore, would be to test it against other lung cancer cell lines. If this proves successful, then the efficiency of this extract against lung carcinomas could be explored.
